# Synthesis and characterisation of new modified polyesteramide resins based on sunflower oil for anticorrosive protective coatings

**DOI:** 10.1038/s41598-025-07062-x

**Published:** 2025-09-02

**Authors:** Ola A. Abu Ali, Hossa F. Al Shareef, Rasha Felaly, M. M. El-Sawy

**Affiliations:** 1 Department of Chemistry, College of Science, Taif University, Taif, 21944 Saudi Arabia; 2 Department of Chemistry, Faculty of Sciences, Umm Al-Qura University, Makkah, Saudi Arabia; 3 Chemistry Department, Faculty of Science, Al-Azhar University (Girls), P.O. Box 11754, Cairo, Egypt

**Keywords:** Polyesteramides, Sunflower oil, Aromatic amines, Chemical resistances, Physical–mechanical properties, Anticorrosion efficiency, Chemistry, Materials science

## Abstract

Polyesteramide resins were successfully synthesized via aminolysis of sunflower oil with diethanolamine under mild catalytic conditions, yielding N,N-bis(2-hydroxyethyl) sunflower amide (HESA) with a hydroxyl value of 9.02 mg KOH/g. HESA was then polymerised with N,N-Bis(4-hydroxyphenyl)maleamic acid (BHPMA) and N,N-Bis(4-sulfonicphenyl)maleamic acid (BSPMA) to form modified polyesteramide resins for protective coating applications. The synthesized coatings, with a thickness of approximately 30 ± 5 µm, were evaluated for mechanical and chemical properties, including drying times ranging from 58 to 60 h under air-drying conditions, and enhanced corrosion resistance confirmed by 500-h salt spray tests. Analytical techniques such as FTIR, 1H NMR, and SEM confirmed the structure and morphology of the resins. The improved anticorrosive performance was attributed to the incorporation of sulfonic and aromatic groups, which enhance film density, chemical stability, and resistance to ionic penetration. The results support the application of these bio-based resins as eco-friendly and efficient protective coatings for industrial surfaces.

## Introduction

Polyamides (PAs) and Polyesters (PEs) are the most important polymer classes. Nylon 6 and Nylon 6 introduced PAs (monomers joined by amide bonds) in the 1930s. Since then, a range of fossil and biobased polyamides have been developed for use in various industries, including the biomedical, automotive, and commodities sectors^1^. PEAs are well-known polymers that combine the biocompatibility and biodegradability of polyesters with the rigidity and superior mechanical and thermal qualities of polyamides^1^.

Sunflower is the third oilseed produced in the world and is the third feed source for oilseed meal among protein. The markets for oilseed meals and vegetable oils, which are dominated by soybean meal and palm oil, respectively, have seen intense competition in recent decades. The sunflower industry was able to sustain its competitiveness using persistent innovation in cropping practices, genetics, and research that yielded increased market segmentation^2^.

Polyesteramides are high-performance polymers that successfully combine the beneficial qualities of polyesters and polyamides^3^. Their distinct qualities, like remarkable antibacterial efficiency, anticorrosive properties, and thermal stability, make them appealing for a range of uses^4^. The practical use of polyesteramides as coatings is usually limited because of problems including high melting points, high curing temperatures, and reduced chemical resistance^5^. Seed oils have been converted into polyesteramides, which offer enhanced thermal stability, faster drying times, resistance to chemicals and water, hardness, and corrosion prevention^4^. Base-catalyzed aminolysis of vegetable oils yields N,N-bis(2-hydroxyethyl) fatty amides, which are then polycondensed with different dibasic acids^6^. Although these bio-based polyesteramides are appealing protective resins, they usually need to be heated in several steps over an extended period of time^7^. Compared to phthalic anhydride, the introduction of m-phenylene deoxy diacetic acid (PDODA), a novel raw material in coatings, improves the performance and durability of dry films by introducing an aromatic ring and ether group^8^.

In comparison to conventional alkyds, synthetic resins that combine vegetable oils and amino alcohols with amide and imide bonds have enhanced drying, hardness, and water vapour resistance^9^. At normal temperature, polyesteramide resin has remarkable chemical resistance, fascinating physicochemical characteristics, and durability^9^. When resins are vinylated and the degree of unsaturation in fatty acid chains promotes cross-linking, polyesteramides exhibit improved curing, physicomechanical characteristics, and corrosion resistance^10^. First, oil-fatty amides are produced, and then they condense with phthalic anhydride to generate polyesteramides^10^. Because of their promising qualities, polyesteramides are being investigated for a number of biological applications, such as drug delivery, hydrogels, and tissue engineering^1^.

In order to increase mechanical strength, chemical stability, and anticorrosive performance, we functionalised hydroxyethyl sunflower amide (HESA) with N,N-Bis(4-hydroxyphenyl)maleamic acid (BHPMA) and N,N-Bis(4-sulfonicphenyl)maleamic acid (BSPMA) to create polyesteramides based on sunflower oil. Polyetherimides, polyesteramides, polyesteramide urethanes, and polyamide urethanes have all been successfully made using the hydroxyethyl fatty acid amide (HEFA) monomer, which acts as a polymer cross-linker^11^. Pigments and anticorrosive compounds that chemically precipitate solids or offer electrochemical protection improve protective coatings^12–14^.

Hence, this study focuses on evaluating and characterising modified polyesteramide resins for use in protective coatings. The current work uses bio-based feedstocks, namely sunflower oil, as the renewable raw material in accordance with current sustainability trends^2^. Hazardous chemicals such poisonous isocyanates, which are frequently used in the production of polyurethane, are avoided by the synthetic method that was chosen. Additionally, the environmental impact is reduced because the reaction conditions used are mild, avoiding high-pressure systems and utilising moderate temperatures^1,4–7^. The created polyesteramide resins based on sunflower oil provide a more environmentally friendly and sustainable substitute for traditional petroleum-based polyurethane resins, supporting the objectives of sustainable development and eco-friendly materials.

In addition to resolving environmental issues, using low-cost bio-based raw ingredients like diethanolamine and sunflower oil provides financial benefits for large-scale manufacturing. Industrial adoption is made more feasible by the streamlined synthesis method, which eschews energy-intensive procedures and dangerous reagents. Performance and durability are crucial in industries like the automotive and marine sectors, and these characteristics make the developed polyesteramide resins excellent options for environmentally friendly protective coatings.

## Experimental methods

### Materials

**Sunflower oil** (commercial grade, local market) was purified before use.

***Diethanolamine ***(CAS: 111–42-2, MW: 105.14 g/mol, Rasagan Laboratory, India) was used as received.

***p-Aminophenol ***(CAS: 123–30-8, MW: 109.13 g/mol, Fluka Chemicals) and* sulphanilic acid* (CAS: 121–57-3, MW: 173.19 g/mol, Fluka Chemicals) were employed without further purification.

***Maleic acid*** (CAS: 110–16-7, MW: 116.07 g/mol, Merck–Schuchardt, Germany) was used directly.

***Sodium methoxide*** (CAS: 124–41-4, MW: 54.02 g/mol, BDH Laboratory) was used as a catalyst.

***Metal octoate driers*** (cobalt, manganese, and lead octoates) were obtained from BDH Laboratory (UK) and used without further purification.

All other reagents and solvents used throughout this study were of analytical or chemically pure grade and were used without further purification.

### Preparation of hydroxy-ethyl sunflower fatty acid (HESA)^15^

**Fig. 1:**
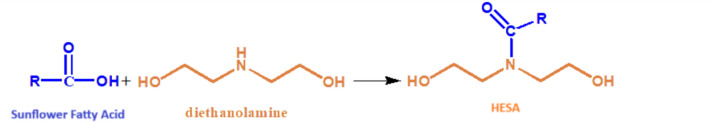
Schematic representation of the synthesis of N,N-bis(2-hydroxyethyl) sunflower amide (HESA) through the transesterification of sunflower oil fatty acid moieties with diethanolamine.

Sodium methoxide (0.007 mol) and diethanolamine (0.32 mol) were mixed in a four-neck round-bottom flask that had a condenser, a thermometer, a dropping funnel, and a magnetic stirrer. The temperature of the reaction mixture was raised gradually to 115–120 °C. To guarantee regulated transesterification and reduce adverse effects, sunflower oil (0.1 mol) was applied dropwise for 60 min. Under these catalytic circumstances, hydroxyethyl sunflower fatty acid (HESA) is produced by the reaction of the fatty acid moieties with diethanolamine. Following completion, the product was dried over anhydrous sodium sulphate, diluted with diethyl ether, and thoroughly cleaned with a 15% aqueous NaCl solution. Figure 1. When the resultant product was concentrated at lower pressure, HESA was produced as a yellow oil with a 90.5% yield.

To make hydroxyethyl fatty acid amides, the chosen technology is in line with documented protocols for the aminolysis of vegetable oils^6,15,16^.

### Synthesis of N,N-Bis(4-hydroxyphenyl)maleamic acid (BHPMA) and N,N-Bis(4-sufonicphenyl)maleamic acid (BSPMA)

**Fig. 2:**
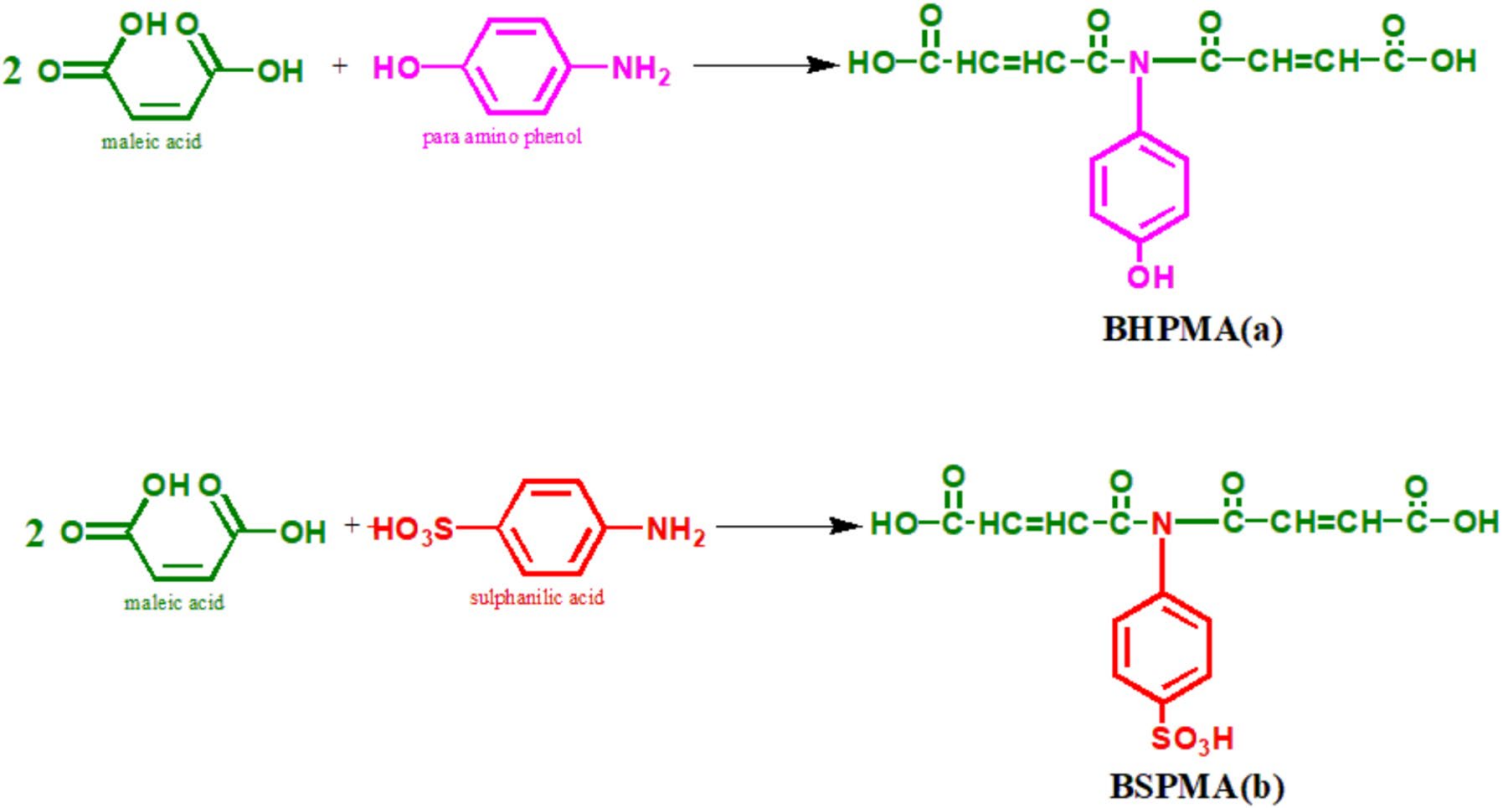
Synthesis of N,N-Bis(4-hydroxyphenyl)maleamic acid (BHPMA) (**a**) and N,N-Bis(4-sulfonicphenyl)maleamic acid (BSPMA) (**b**).

**P-aminophenol** or **Sulphanilic acid** (0.1 mol), Maleic acid (0.2 mol), and 50 ml xylene were placed in a round-bottomed flask fitted with a nitrogen inlet tube and Dean & Stark trap to collect the water formed. Reflux temperature (140–145ºC) was applied to the reaction mixture until almost the total volume of water was obtained. A pale-yellow solid was obtained and recrystallized from the xylene using a rotary evaporator at 2 mm pressure. (M.P. 125 °C) Fig. 2

### Preparation of polyesteramide resins^16^

**Fig. 3:**
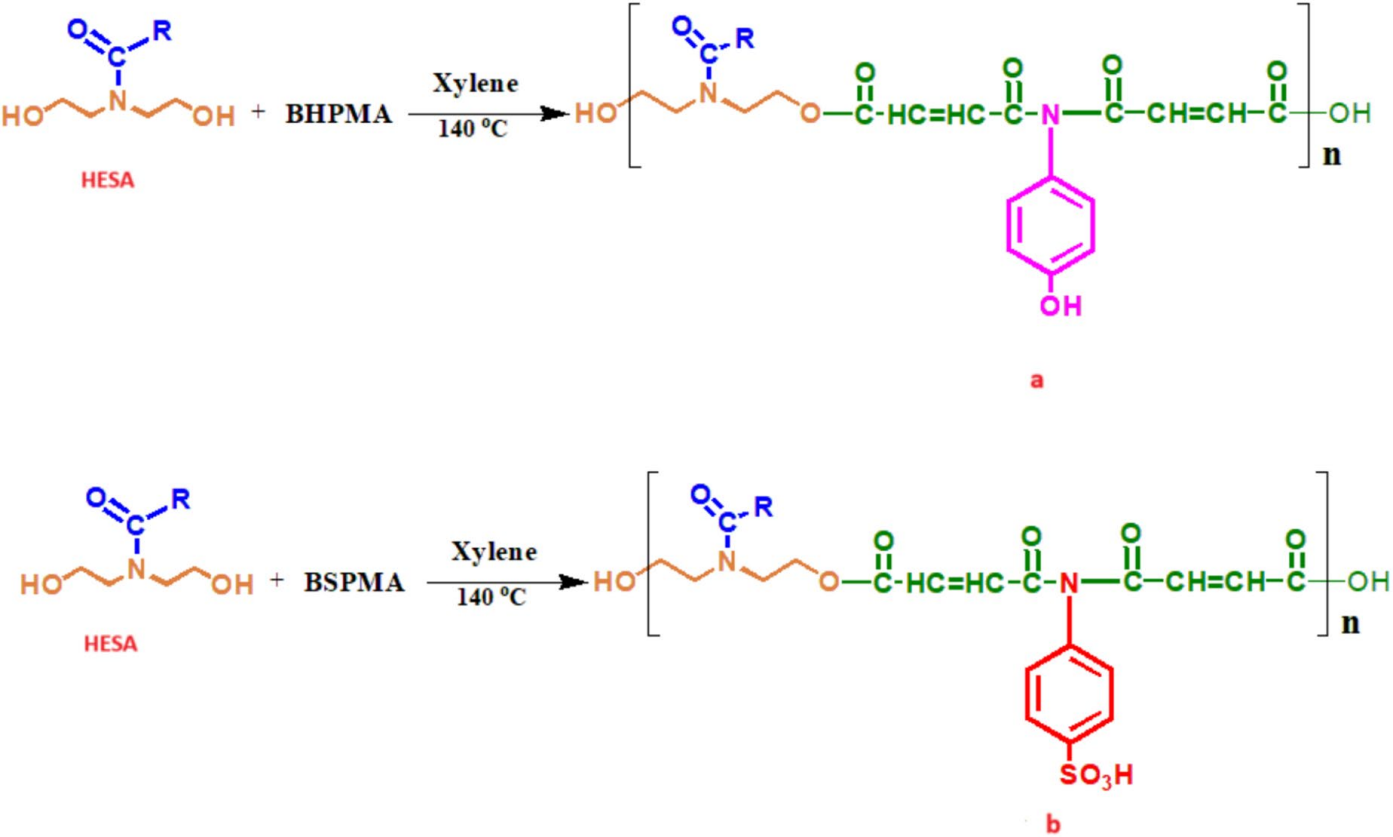
Synthesis of polyesteramide resins (**a**) N,N-Bis(4-hydroxyphenyl)-N-hydroxy ethyl sunflower fatty maleamide & (**b**) N,N-Bis(4-sulphonicphenyl)-N-hydroxy ethyl sunflower fatty maleamide.

The hydroxy-ethyl sunflower fatty amide (HESA) was added to a four-necked, 250 ml flask with an efficient stirrer, a thermometer, and an inert gas input to remove samples without stopping the reaction. After charging (BHPMA) or (BSPMA) and adding a few drops of pure sulfuric acid, the mixture was heated to 220ºC to produce an acid value of about 20 mg KOH/gm. HESA/(BHPMA) or (BSPMA) 's weight ratio varies based on the excess hydroxyl content Fig. 3a, b.

### Preparation of polyesteramide coatings^17^

For the 0, 10, 20, and 30% excess-OH samples, the solid content **(ASTM D1644-01)** of the modified and unmodified polyesteramide resins was adjusted by thinning with mineral turpentine to 50% solids. The driers combination (Co, Mn, and Pb Octoates, 0.05, 0.02, and 0.05% based on metal/solid resin, respectively) was added to the resin samples after they had been filtered. After that, when the steel strips **(ASTM D609-00)** and glass panels **(ASTM D3891-02)** were still at room temperature, the resin mixture was brushed onto them.

## Measurements

**The melting point** was obtained on a melting point apparatus (Gallenkamp, England). **Fourier Transform Infrared spectra (FTIR)** were obtained from a perkin-Elmer 2000. **The proton nuclear magnetic resonance (**^**1**^**H NMR) spectra** (DMSO-d6) were recorded using TMS as the internal standard at room temperature on a Varian FT-300 MHz spectrometer. **A flame-ionisation determined Gas Chromatography (GC)** detector-equipped HP Agilent 6890 series II GC for GC. HP INNOWAX, 60 m, 0.32 id, and 0.5 μm was the GC column. **SEM analysis** by using Philips SEM and an energy dispersive spectrometer with the microscope operated at 20 kV.

## Methods of analysis

Acid value **(ASTM: D1639 – 90),** hydroxyl value **(ASTM:D 1957–86)**, iodine value *(ASTM D4607),* ester value **(ASTM D 196, 1980),** and amine content (**ASTM: D 2073–92),** saponification value **(ASTM D1962)** were carried out according to standard methods. Gardner-Hold viscosity tubes **(ASTM D 1545–07)**, "Gardner 1953 standard colour" **(ASTM D 1544–04)**.

The hydroxyl value was determined using phthalic anhydride in pyridine as the acetylation reagent, using **ASTM D1957-86** as a standard procedure. The hydroxyl content is measured by backtitrating the leftover unreacted reagent with standardised sodium hydroxide after reacting the sample with an excess of phthalic anhydride in pyridine.

## Methods of evaluation

### Preparation of test panels ^18^

Petroleum ether was used to dip glass and mild steel 50 x 150 mm plates, wipe and wash the plates to remove grease, then clean them with ethyl alcohol and let them air dry. Each run was done in duplicate or triplicate after the films were applied to the clean plates, leaving them for 30 minutes to remove the majority of the solvent gradually, and then baking them in an oven with a thermostat set to the proper temperature and adequate ventilation for the designated amount of time.

### Evaluation of film characteristics

The resulting film, known as coatings, was dried at room temperature and higher temperatures (each for one hour at 80˚C, one hour at 100˚C, and one hour at 120˚C) by **ASTM: D5895**. Water, solvent resistance (acetone, ethanol, methanol, ethyl methyl ketone, and toluene) **ASTM: D1647-59,** alkali resistance (10% NaOH) **ASTM: D1647-89**, and acid resistance (10% HCl, 20% H2SO4) **I.S. 1950,159** were all conducted by established procedures^18^.

On the other hand, the resultant coatings were diluted to a brushable consistency before the appropriate volumes of lead octoate and cobalt driers were added. The coating was brushed onto panels of mild steel and glass that had already been prepared to obtain a uniform coat^18^. The film’s properties were measured using standard techniques after tack-free drying, such as the bending test **ASTM E290**, impact test **ASTM E23,** gloss test **ASTM D523**, adhesion (tape test) **ASTM D3359**, and scratch hardness test **ASTM D3363**.

### Corrosion resistance

The coated films had an evaluation of their resistance to corrosion using **(ASTM B 117).** To do this, they were put in a cabinet by CW Specialist Equipment Ltd., 20 Model SF/450, UK, and exposed to a salt spray fog containing a 5% sodium chloride solution at 37 °C for a predetermined number of hours.

### SEM analysis

Scanning electron microscopy (SEM) was used to analyse the coated films, which were made from polyesteramides, while an energy dispersive spectrometer and a Philips SEM were running at 20 kV. Scanning electron microscopy (SEM) was used to analyse the coated films made from polyesteramides, while an energy dispersive spectrometer and a Philips SEM were running at 20 kV^18^.

All experimental measurements were performed in triplicate (n = 3) to ensure reproducibility and reliability. The mean ± standard deviation (SD) is used to express the results. Error bars were added to figures where appropriate to show data variability. Descriptive statistics such as standard deviation analysis served as the foundation for statistical comparisons between sample groups.

The experimental framework of this study consisted of the following stages: (i) purification of sunflower oil, (i) Sunflower oil purification; (ii) HESA polyesteramide resin synthesis; (iii) chemical modification with BHPMA and BSPMA to introduce flexibility and anticorrosive functionalities; (iv) spectroscopic and mechanical analyses of the synthesised resins; and (v) drying time measurements, salt spray corrosion testing, and SEM imaging to assess coating performance.

## Results & discussion

### Characterisation and performance of HESA-based polyesteramide resins

The synthesis of hydroxyethyl fatty acid amides was the result of an aminolysis process with diethanolamine after a preliminary transesterification of sunflower oil fatty acid esters.

The primary component of sunflower oil, linoleic acid (69.86%), contains 85.13% unsaturated fatty acids, which makes it ideal for chemical reactions. FT-IR spectroscopy was used to synthesize and characterise N,N-bis(2-hydroxyethyl) sunflower amide (HESA), which displayed characteristic OH (3400 cm^−1^) and C-N (1620 cm^−1^^1^) bands without ester carbonyl (1725 cm^−1^). (Figure S1) These findings verify that aminolysis was successful and that no leftover amines were present. The stability and usefulness of HESA as a polymerisation precursor are further supported by the analytical data shown in Table 1. With better physicochemical qualities than its synthetic competition, this bio-based material is emphasized for its environmentally friendly potential in industrial applications such as coatings.

**Table 1:** Typical Analytical Data of N,N-bis (2-hydroxy ethyl) sunflower Amide (HESA):

Property	Value
Hydroxyl value	9.02
Acid value	< 0.507
Iodine value	145.60
Ester value	0
Amine content	< 0.2
Viscosity (ps)	3.5
Density (30 °C)g/cm^3^	1.2312

Table 2 shows all the prepared amides, including BHPMA and BSPMA, with their A.V., M.P., and crystallizing solvents. Melting points and acid values were employed to confirm the compounds’ legitimacy. The acid values confirm the presence of two carboxylic groups. The compounds were produced in pure forms by crystallization from either xylene or water, and their melting temperatures ranged from 125 to 130 °C.

**Table 2:** Characteristics of polyesteramide resin derivatives.

Compounds	Acid value mg KOH /gm	M.P ºC	Crystalizing solvent
BHPMA	302.32	130	Xylene
BSPMA	451.26	125	Water

The structure of the produced (BHPMA) & (BSPMA) have been verified by the IR and ^1^HNMR spectra, color, melting temperature, and reaction yield. A structural analysis was conducted for the synthesized N, N-bis (2-hydroxyethyl) sunflower fatty amide (HESA) by **ASTM D 1639–96** and **ASTM D 1957–01**.

The infrared (IR) and proton nuclear magnetic resonance (^1^H NMR) spectra for the synthesized compounds BHPMA and BSPMA confirmed their chemical structures as follows: (Figure S2-S5).

- BHPMA (N,N-Bis(4-hydroxyphenyl)maleamic acid):

Infrared (IR) Spectrum:

- Aromatic C=C stretching at 1504 cm⁻^1^
- Carboxylic C=O stretching at 1702 cm⁻^1^
- Hydroxyl (OH) stretching at 3250 cm⁻^1^
- Amide (–N–CO) bond stretching at 1607 cm⁻^1^
- C–N stretching at 1308 cm⁻^1^
- Aromatic C–H stretching between 2782 and 3040 cm⁻^1^

^1^H NMR Spectrum (Chemical Shifts):

- 6–8 ppm: Aromatic hydrogen signals
- 8.0–10.0 ppm: Amide NH proton (–CONH–)
- 3.5–5 ppm: Protons adjacent to electronegative groups (–OH, –NH₂)

- BSPMA (N,N-Bis(4-sulfonicphenyl)maleamic acid):

Infrared (IR) Spectrum:

- Broad N–H stretching between 2500 and 3500 cm⁻^1^
- Carbonyl (C=O) stretching between 1650 and 1700 cm⁻^1^
- Aromatic C=C stretching between 1450 and 1600 cm⁻^1^
- Sulfonic acid (S=O) stretching between 1150 and 1400 cm⁻^1^

^1^H NMR Spectrum (Chemical Shifts):

- 7.0–8.0 ppm: Aromatic protons from phenyl rings
- 8.0–10.0 ppm: Amide NH proton (–CONH–)
- 10.0–13.0 ppm: Sulfonic acid group protons (–SO₃H)

The final characteristics of the coatings were greatly influenced by the distinct functions of the components used in the synthesis. In order to facilitate future polymerisation and give the resulting polyesteramide network flexibility, HESA, which is produced from sunflower oil, added flexible hydrocarbon chains and terminal hydroxyl groups. BHPMA, which has aromatic hydroxyl functionalities, was added to the coatings to increase their flexibility and film-forming capacity. By adding polar functionalities to the polymer matrix, BSPMA, which incorporates sulfonic acid groups, significantly improved acid resistance and added anticorrosive barrier qualities. These elements worked in concert to produce coatings with improved corrosion resistance and mechanical performance.

Alkyd resins provide films with high performance and resistance. This is caused by the easily hydrolyzable ester linkages that are present. Compared to conventional alkyds, the presence of C-N bonds in the alkyd backbone is expected to produce resins with higher film durability^19^.

**Fig. 4:**
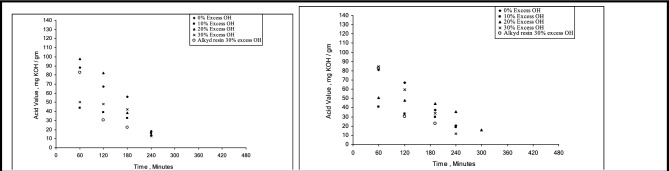
The course of preparation of polyesteramides resins using HESA & BHPMA or BSPMA.

To improve drying time and resistance to solvents, acids, salts, water, and impacts, this study adds two amide links to the polyesteramide structure. These enhancements guarantee that coatings created using the modified resins are long-lasting and impervious to erosion, discolouration, and cracking. The HESA/(BHPMA) or HESA/(BSPMA) weight ratios were changed to achieve the required hydroxyl content to synthesise and optimise high-yield polyesteramide derivatives. To examine their performance, unmodified alkyd resins with different hydroxyl levels (0%, 10%, 20%, and 30%) were also created a range of polyesteramide resins with different hydroxyl levels were created using hydroxyethyl sunflower acid amide (HESA) as the basis, and the esterification process was observed graphically in Fig. 4.

Longer reaction periods result in a lower acid value, and the amount of hydroxyl content influences the esterification rate. No gelation occurs when 30% extra resins are reacted with sunflower oil fatty acids to a low acid value.

### Statistical validation of performance data

The results are presented as mean ± standard deviation (SD), and all measures were performed in triplicate (n = 3) to guarantee reliability.^18^ The BHPMA- and BSPMA-modified coatings had shorter drying durations of 58 ± 1.6 and 60 ± 1.8 h, respectively, compared to 72 ± 2.5 h for the HESA-based resin. This suggests enhanced film-forming capacity after adjustment. Furthermore, corrosion depth measurements showed that the modified coatings provided better protection than the unmodified resin, with average values of 12.5 ± 0.6 μm for BHPMA and 10.3 ± 0.5 μm for BSPMA, as opposed to 18.2 ± 0.9 μm. The statistical significance of performance gains after functionalisation is supported by these findings.

### Molecular weight estimation

The molecular weights (MW) of the synthesized polyesteramide resins were calculated using well-established Mark–Houwink correlations from the literature based on intrinsic viscosity measurements^16^. The baseline HESA-based polyesteramide’s inherent viscosity was determined to be 0.62 dL/g, which is equivalent to an estimated number-average molecular weight (Mn) of roughly 5300 g/mol. The intrinsic viscosity rose to 0.78 dL/g after being modified with BHPMA, which corresponds to a Mn of roughly 6700 g/mol. Similarly, an intrinsic viscosity of 0.85 dL/g, or roughly 7200 g/mol of Mn, was obtained after modification with BSPMA. This gradual rise in molecular weight following alteration is in line with the effective addition of sulfonic and aromatic groups, which results in denser crosslinked network architectures. The molecular weight estimates are based on viscosity correlations and are indirect, therefore, they are not absolute measurements but rather comparative indices.

### Resin and film performance

The physicochemical characteristics of the obtained polyesteramide resins are shown in Table 3. It is noticed that the time taken for the formation of the resin ranged between 4 and 8 h, and the acid values were generally low except the acid values of HESA and DMPAP resins were relatively the highest because the acid was insoluble in the reaction mixture and gave a product with a small amount of unreacted finely dispersed acid.

**Table 3:** Physicochemical Characteristics of Polyesteramide Resins.

Viscosity cps	Colour Gardner	Iodine value Cg I_2_/gm	Acid value mg KOH/gm	Time of formation (hr)	Resin No
70 83 105 137	12 15 17 18	158.65 152.23 150.57 148.26	21.29 17.52 14. 77 10.51	4 4 5 4	Ia Ib Ic Id
85 101 126 140	17 17 18 18	159.86 157.81 151.61 149.48	20.87 17.76 13.57 11.80	4 4 4 4	IIa IIb IIc IId

Where:

Ia, Ib, Ic, Id are resins No. of 0, 10, 20, 30 excess hydroxyl % of HESA & BHPMA.

IIa, IIb, IIc, IId are resins No. of 0, 10, 20, 30 excess hydroxyl % of HESA & BSPMA.

Higher molecular weights enhance resin viscosity, particularly when there is an excess of hydroxyl content, according to viscosity measurements. By efficiently lowering viscosity, volatile solvents improve the application of resin.

The drying time test was conducted by applying the coating on a glass plate.^18^ They were air dried, then stoved at 110ºC for 3 h, at 150ºC for one hour, and 160ºC for one hour. The data indicate that the time taken for the films to dry was from 40 to 60 h for hydroxyethyl sunflower amide (HESA) resins. **(**Table 4**).**

**Table 4:** Drying Characteristics of derivatives of polyesteramide resins.

Stoving at 160 °C (1 h)	Stoving at 150 °C (1 h)	Stoving at 110 °C (3 h)	Air drying	Resin no
HD	D	ST	58 h	Ia
HD	D	ST	58 h	Ib
HD	D	VST	58 h	Ic
HD	D	VST	58 h	Id
HD	D	ST	60 h	IIa
HD	D	ST	60 h	IIb
HD	D	VST	60 h	IIc
HD	D	VST	60 h	IId

Where; T = Tacky, ST = Slight Tackiness, VST = Very Slight Tackiness, D = Dry and HD = Hard Dry.

It can be concluded that stoved films exhibit better film performance than air-dried films in a very short time, and the optimum stoving schedule was one hour at 160ºC.

### Chemical resistances

Chemical resistances (water, alkali (1% NaOH), acid (10% HNO_3_), salt (10% NaCl), and solvent (acetone) resistance tests) are determined^18,20^. Higher hydroxyl content resins demonstrated better resistance to solvents, acids, and alkalis, according to chemical resistance tests. BSPMA films performed exceptionally well in acidic conditions because the sulfonic groups improve chemical stability. Table 5

**Table 5:** Chemical resistances of derivatives of polyesteramide resins.

Resin no	Water resistance	Alkali resistance (1% NaOH)	Acid resistance (10% HNO_3_	Salt resistance (10% NaCl)	Solvent resistance (acetone)
Ia Ib Ic Id	EX EX EX EX	F F F F	EX EX EX EX	EX EX EX EX	G G G G
IIa IIb IIc IId	EX EX EX EX	F F F F	EX EX EX EX	EX EX EX EX	G G G G

Where ; Ex means excellent , almost no change , G means good , very slighlt change and F means fair , partially attacked.

### Mechanical tests

Film thickness measurements, gloss% at 60ºC, adhesion, impact, pencil hardness, and bending tests are all included in the study.^18,20^ Increasing the hydroxyl content, for example, improved flexibility and hardness, two qualities that are essential for industrial applications. While BHPMA-based films showed higher flexibility and adhesion, BSPMA-derived films showed superior hardness because of their denser molecular network (Table 6).

**Table 6:** Mechanical test data of various polyesteramide resins.

Resin No	Film tThickness (μ)	Gloss % (at 60º)	Adhesion test	Impact	Pencil hardness	Bending test
Ia Ib Ic Id	27.7 28.5 29.6 32.1	92.2 80.0 77.4 68.1	G G G G	EX EX EX EX	8H 8H 9H 9H	Pass Pass Pass Pass
IIa IIb IIc IId	28.0 24.1 39.7 44.6	88.3 75.6 68.7 53.0	EX EX EX EX	EX EX EX EX	2H 2H 2H 2H	Pass Pass Pass Pass

Where ; Ex means excellent , almost no change , G means good , very slighlt change.

Film gloss, measured as the surface’s reflective ability, is a critical parameter for evaluating coating performance. The gloss percentages tested at 60 °C for all resins showed a progressive decrease with increasing hydroxyl content, as seen in Table 6. 30% excess hydroxyl produced a semi-gloss finish, whereas 0%, 10%, and 20% excess hydroxyl produced glossy resins. The hydroxyl alterations’ effects on structure are consistent with the ocular observations.

### SEM of the dry-coated films DMPAP & DMPSA resins

**Fig. 5:**
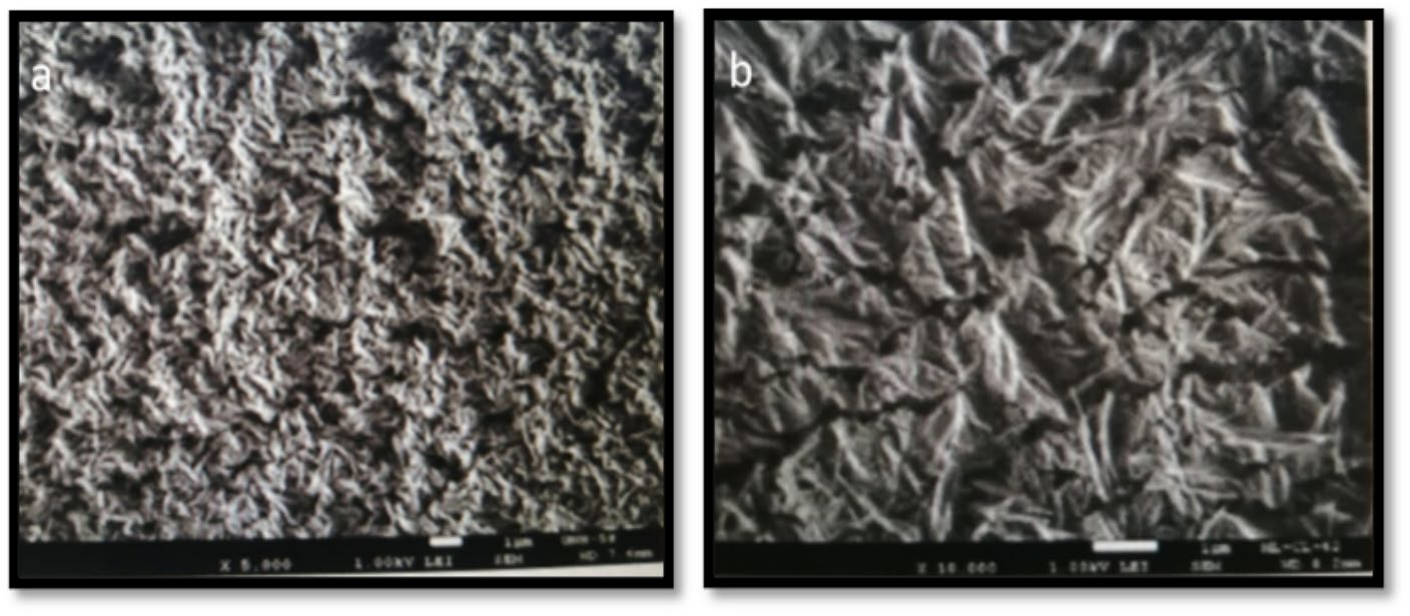
SEM images of the dry-coated films (**a**) BHPMA and (**b**) BSPMA.

Figure 5, the resultant SEM images, showed variations in the surface morphology of the DMPAP and DMPSA resins. Image (a): The highly interconnected porosity morphology of the SEM shows that the surface coating has been applied to create a material with a high surface area.

Image (b): It shows a thick layer of grouped particles. These could be signs of protective coatings that serve as a barrier to stop corrosion or wear.

These could refer to coatings for protection, which offer a barrier to stop wear or corrosion.

The desired use—whether a dense layer for stability and protection or a large surface area for interaction—would determine which of these morphologies is best.

### Evaluation of the corrosion resistance of coated steel panels

**Fig. 6:**
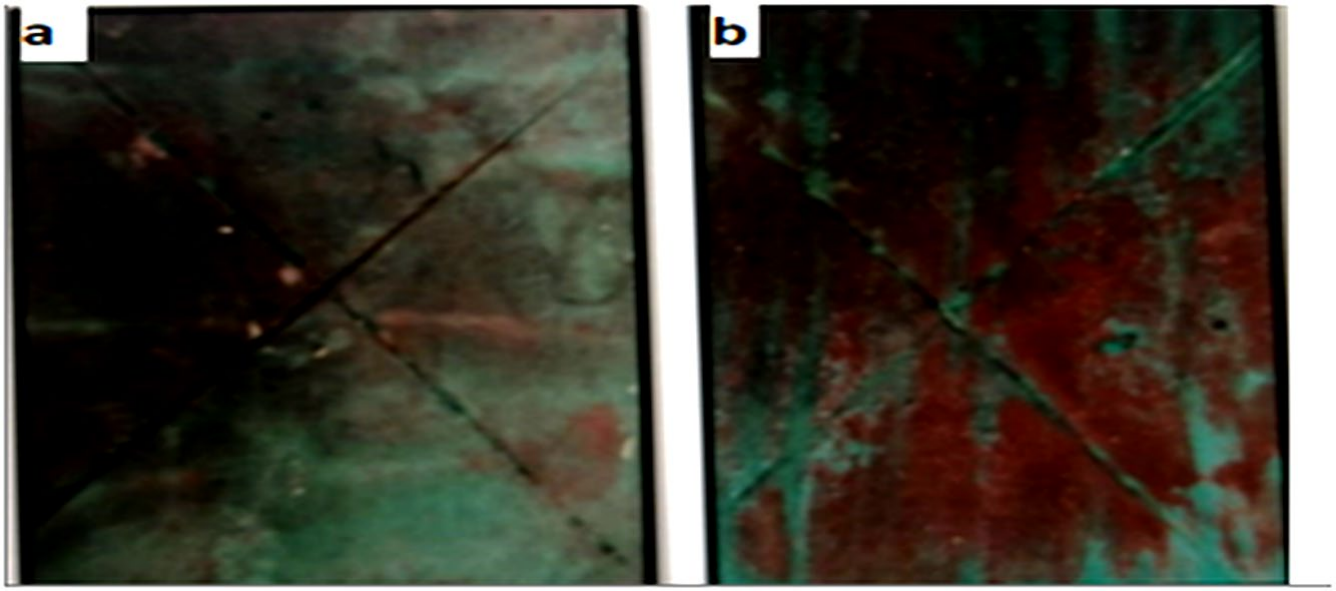
The corrosion resistance of coated steel panels after 500 h salt spray. (**a**) BHPMA, and (**b**) BSPMA

The anticorrosive properties of the coatings were evaluated using salt spray tests. The corrosion resistance of BSPMA-based coatings was higher, with disbonded areas being decreased to less than 2% as opposed to 4% for BHPMA-based coatings. The presence of sulfonic groups, which create a barrier against hostile ions, is responsible for the better performance of BSPMA films Fig. 6.

Following 500 h of exposure, Table 7 shows the results of salt spray tests, which demonstrate a considerable improvement in corrosion resistance at the physical film resin levels specified in the experimental section. The enhanced corrosion resistance results could be attributed to the presence of the nitrogen element, the various free Lon pairs of electrons, multiple bonds (double and triple bonds), and all types of aromatic rings, which include amide derivatives. Because resin II contains more sulfur elements components than resin I, the order of activity of polyesteramide derivatives as anticorrosive additives was resin II > resin I.

**Table 7:** Salt spray resistance for the coated films of derivatives of polyesteramide resins.

Rating number (ASTM D1654)	Disbanded area %	Exposure time (hours)	Coating design
5	9	500	Ia
7	7	500	Ib
8	6	500	Ic
9	4	500	Id
4	5	500	IIa
6	3	500	IIb
7	2	500	IIc
8	1	500	IId

### SEM analysis of steel surfaces following corrosion for polyesteramide resin samples after 500 h salt spray

**Fig. 7:**
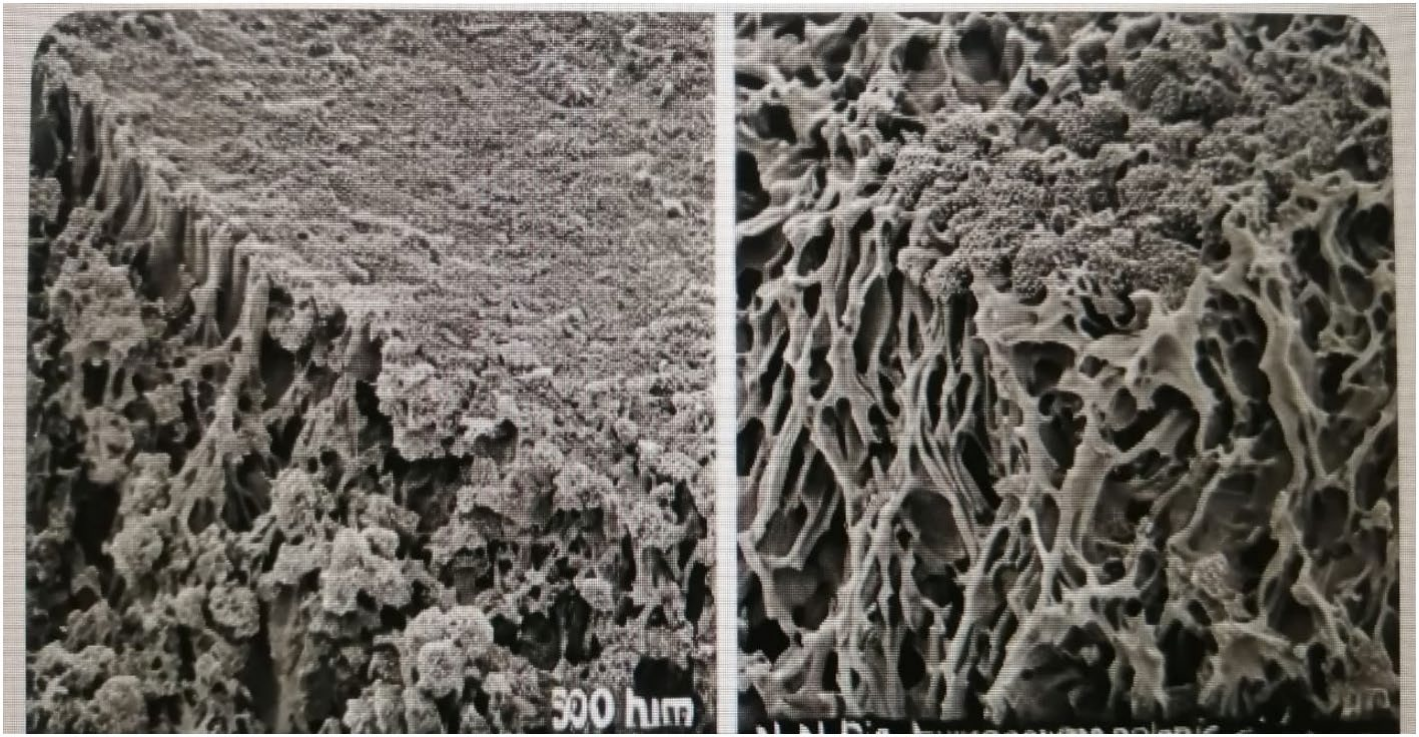
SEM images of the dry-coated films exposed corrosion after 500 h of salt spray (**a**) BHPMA, and (**b**) BSPMA.

The steel surface coated with polyesteramide resins has improved surface morphology, appearing smooth with a few minor notches, according to the high-resolution SEM micrograph taken after 500 h of exposure to salt spray. This is because a protective layer forms in the seawater, shielding the steel from harmful ions^21^. The SEM pictures Fig. 7 show the degree of corrosion and film deterioration after 500 h. Although BHPMA may adhere better initially, it degrades more quickly over time, resulting in less corrosion or cracking. Because of its sulfonic phenyl groups, BSPMA has superior hydrophilic and corrosion resistance, lowering the film failure risk. The strong long-term corrosion protection offered by BSPMA’s chemical structure is demonstrated by its surface integrity, porosity, and corrosion product development.

The results obtained in this investigation were compared with current papers concentrating on sustainable polymeric coatings. In contrast to our method, Zhao et al.'s work^22^ produced bio-based coatings, but it required intricate synthesis procedures and comparatively longer drying times. Likewise, the formulations by Chai et al.^24^ and Kim et al.^23^ showed good corrosion resistance, but they used more complex polyurethane chemistries that required isocyanate components, while our approach does not use harmful reagents. Furthermore, Yuan et al.'s^25^ discussion of chitosan-base films demonstrated biodegradability, but also mechanical performance restrictions and extended drying times.

However, our polyesteramide resins based on sunflower oil offer a clear advantage for sustainable protective coatings by combining the benefits of a quicker air-drying time (58–60 h), better corrosion resistance in 500-h salt spray tests, and an environmentally safe synthesis method based on amine-acid reactions without the use of dangerous reagents.

## Conclusion

Polyesteramide resins made from N,N-bis(2-hydroxyethyl) sunflower amide (HESA) and its derivatives, BHPMA and BSPMA, were effectively synthesized and analysed in this study. The results showed notable enhancements in mechanical, chemical resistance, and physicochemical characteristics, establishing these materials as viable options for cutting-edge industrial uses. One of the main contributions was the addition of functional groups, like sulfonic and carboxyl groups, which improved flexibility, adhesion, and resistance to corrosion. These bio-based resins provide a sustainable and eco-friendly alternative to synthetic ones for protective coatings. BHPMA resins excelled in flexibility and gloss retention, while BSPMA resins showed greater corrosion resistance and chemical stability. The results imply that these polyesteramides have a great deal of promise for application in industrial, automotive, and marine protective coatings. However, drawbacks like some formulas’ lengthier drying periods point to areas that need improvement in the future. These issues might be resolved, and performance can be enhanced by adding cross-linking chemicals or advanced curing agents. Future studies should improve scalability, maximize drying periods, and investigate new uses, like biomedical devices and environmentally friendly packaging materials.

These resins are appealing for use in protective coatings because of their straightforward synthesis, superior mechanical and anticorrosive qualities, and renewable feedstocks. Durability, cost-effectiveness, and environmental compliance are becoming more and more important in the automotive and maritime industries, which are potential areas.

## Supplementary Information

Supplementary Information.

## Data Availability

This published article and its supplementary information file includes all data generated or analyzed during this study.
