# Supplementary material for: Synthesis and characterisation of new modified polyesteramide resins based on sunflower oil for anticorrosive protective coatings
Source: Sci Rep. 2025 Sep 2;15:32265. doi: 10.1038/s41598-025-07062-x (PMC12402498; doi:10.1038/s41598-025-07062-x)
Supplement: Supplementary file 1 — Supplementary Information. [file 41598_2025_7062_MOESM1_ESM.docx]

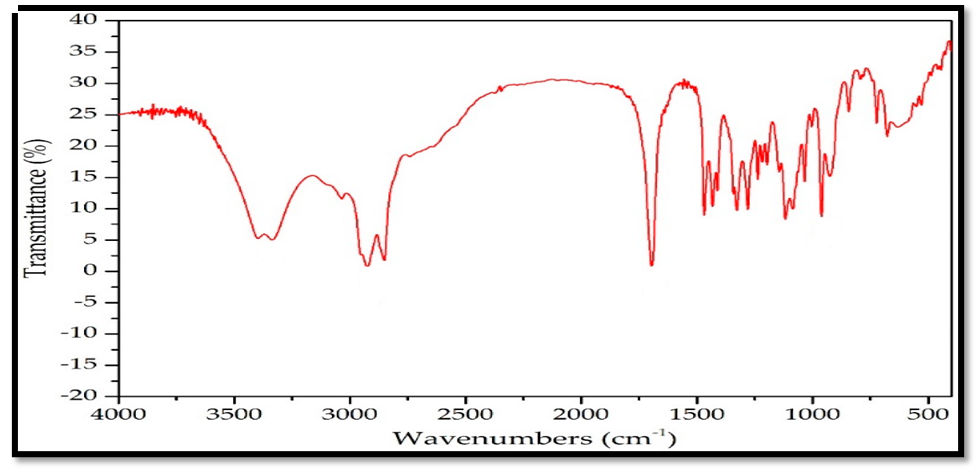


***Fig. S1. IR Spectrum of Hydroxy - Ethyl Sunflower Fatty Acid (HESA)***


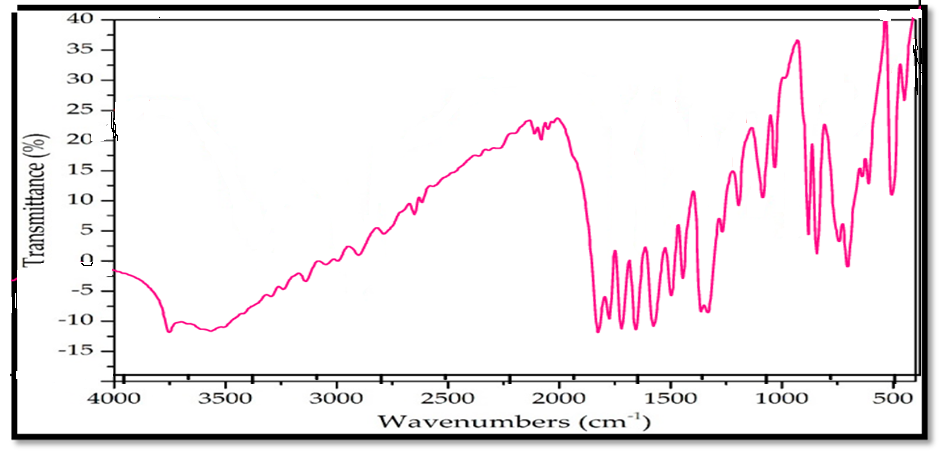


Fig.S2. IR Spectrum of BHPMA


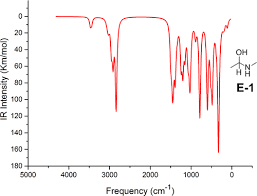


Fig.S3. IR Spectrum of BSPMA


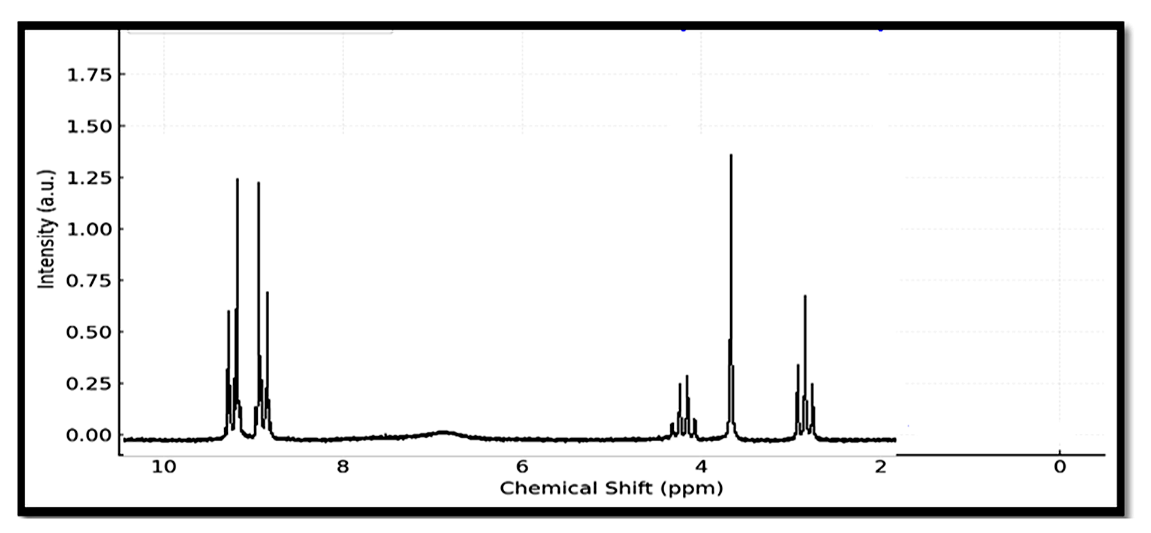


Fig. S4. NMR Spectrum of BHPMA


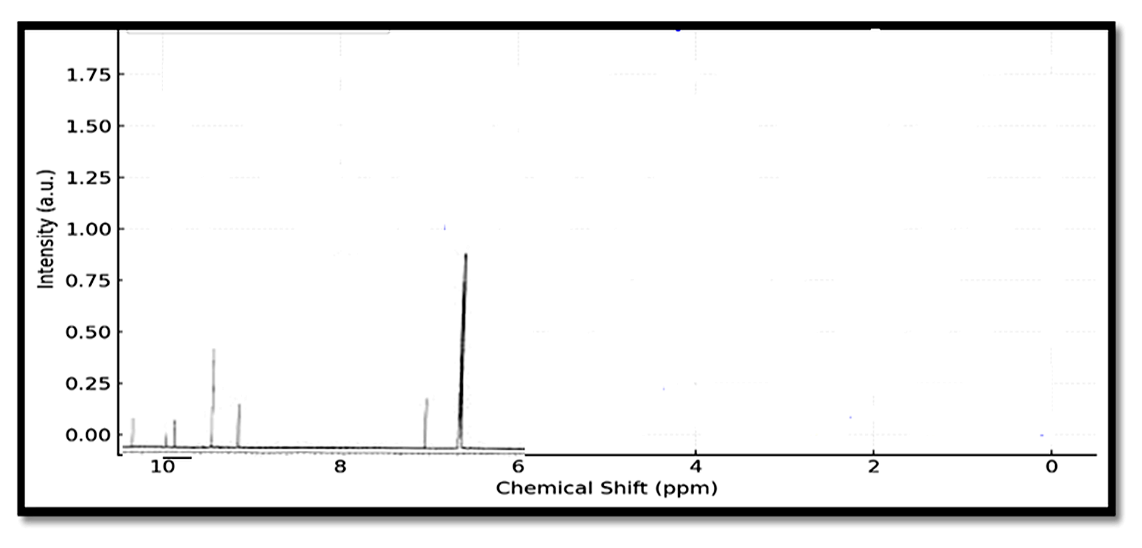


Fig. S5. NMR Specrtum of BSPMA
